# *Mycobacterium bovis* BCG promotes tumor cell survival from tumor necrosis factor-α-induced apoptosis

**DOI:** 10.1186/1476-4598-13-210

**Published:** 2014-09-11

**Authors:** Sahana Holla, Devram Sampat Ghorpade, Vikas Singh, Kushagra Bansal, Kithiganahalli Narayanaswamy Balaji

**Affiliations:** Department of Microbiology and Cell Biology, Indian Institute of Science, Bangalore, 560012 India

**Keywords:** p53, TNF-α, Apoptosis, COP1, SHH signaling, BCG immunotherapy

## Abstract

**Background:**

Increased incidence of lung cancer among pulmonary tuberculosis patients suggests mycobacteria-induced tumorigenic response in the host. The alveolar epithelial cells, candidate cells that form lung adenocarcinoma, constitute a niche for mycobacterial replication and infection. We thus explored the possible mechanism of *M. bovis* Bacillus Calmette-Guérin (BCG)-assisted tumorigenicity in type II epithelial cells, human lung adenocarcinoma A549 and other cancer cells.

**Methods:**

Cancer cell lines originating from lung, colon, bladder, liver, breast, skin and cervix were treated with tumor necrosis factor (TNF)-α in presence or absence of BCG infection. p53, COP1 and sonic hedgehog (SHH) signaling markers were determined by immunoblotting and luciferase assays, and quantitative real time PCR was done for p53-responsive pro-apoptotic genes and SHH signaling markers. MTT assays and Annexin V staining were utilized to study apoptosis. Gain- and loss-of-function approaches were used to investigate the role for SHH and COP1 signaling during apoptosis. A549 xenografted mice were used to validate the contribution of BCG during TNF-α treatment.

**Results:**

Here, we show that BCG inhibits TNF-α-mediated apoptosis in A549 cells via downregulation of p53 expression. Substantiating this observation, BCG rescued A549 xenografts from TNF-α-mediated tumor clearance in nude mice. Furthermore, activation of SHH signaling by BCG induced the expression of an E3 ubiquitin ligase, COP1. SHH-driven COP1 targeted p53, thereby facilitating downregulation of p53-responsive pro-apoptotic genes and inhibition of apoptosis. Similar effects of BCG could be shown for HCT116, T24, MNT-1, HepG2 and HELA cells but not for HCT116 p53^-/-^ and MDA-MB-231 cells.

**Conclusion:**

Our results not only highlight possible explanations for the coexistence of pulmonary tuberculosis and lung cancer but also address probable reasons for failure of BCG immunotherapy of cancers.

**Electronic supplementary material:**

The online version of this article (doi:10.1186/1476-4598-13-210) contains supplementary material, which is available to authorized users.

## Background

The type II alveolar epithelial cells exhibit significant functions during mycobacterial pathogenesis. Along with the alveolar macrophages, they are reservoirs for the pathogenic mycobacteria in the lungs; a site suitable for mycobacterial replication [1, 2]. Additionally, these epithelial cells secrete several cytokines and chemokines that orchestrate the early host immune responses and thus establish the intra-alveolar cytokine network [3]. One of the early cytokines that could regulate epithelial responses during mycobacterial infection is tumor necrosis factor (TNF)-α [4]. TNF-α is a multifunctional cytokine that is significant for activation of macrophages and dendritic cells, granuloma formation and recruitment of lymphocytes to the infection foci during infection [5]. Of note, TNF-α displays potent anti-tumorigenic properties and thus used in the treatment of metastatic melanomas, primary or metastatic unresectable liver tumors and soft tissue sarcomas [6, 7].

In this context, a large percentage of the patients with pulmonary tuberculosis are at high risk for lung cancer, usually adenocarcinomas [8, 9]. Many studies have linked carcinogenesis of the lung tissues to the genetic damage caused by infection-induced inflammation and fibrosis [10, 11]. However, the exact cause and link between the coexistence of lung adenocarcinomas and pulmonary tuberculosis remains obscure.

One of the significant functions of the tumor suppressor p53 is to execute the apoptotic program of the cell [12]. Hence, p53 and p53-dependent regulatory network is the most common deregulated link in many human cancers including the lung cancers [13]. Transactivation properties of p53 during induction of apoptosis results in the expression of multiple apoptotic genes like BH3-only family *PUMA*, *NOXA*, *BID* and Bcl-2 family *BAX* [12]. Interestingly, the turnover of p53 protein is crucial to determine the cell-fate and is tightly regulated by multiple E3 ubiquitin ligases and the proteasome machinery [14, 15]. Various cues including treatment with cytokines like TNF-α induces the transcriptional activation and stabilization of p53 [16].

Several signaling pathways including sonic hedgehog (SHH) signaling, regulate the cellular homeostasis. SHH signaling exhibits myriad functions during embryonic development, wound healing, tissue and organ development and infections [17]. Evidently, deregulated SHH signaling is often associated with many human cancers [18]. Canonical SHH signaling involves its binding to the receptor, Patched-1 (PTCH1), alleviating the inhibition on Smoothened (SMO). Subsequently, SMO leads to the activation of GLI family of transcription factors (GLI1 and GLI2). While inhibitory complex comprising of GSK-3β is inactivated, GLI1 repressor NUMB is degraded. Thus, GLI1 is now functional to transactivate the responsive genes [19].

In the current investigation, we have explored the possible mechanism of mycobacteria-assisted tumorigenicity in type II epithelial cells, A549 human lung adenocarcinoma. Inhibition of TNF-α-induced apoptosis was identified as the mechanism of BCG action as assessed in A549 and several other tumor cells. Moreover, we found that BCG activated the SHH signaling to express an E3 ubiquitin ligase, constitutively photomorphogenic 1 (COP1)/RFWD2 which targets TNF-α-responsive p53 and thus subdues apoptosis. Classical xenograft studies in nude mice showed that BCG suppressed the TNF-α action of tumor clearance; rather promoted tumor formation. Altogether, our results provide the molecular basis of inhibition of apoptosis, executed by mycobacteria in several tumors to promote tumorigenesis.

## Results

### BCG targets p53 to rescue TNF-α-mediated cell death

**Figure 1:**
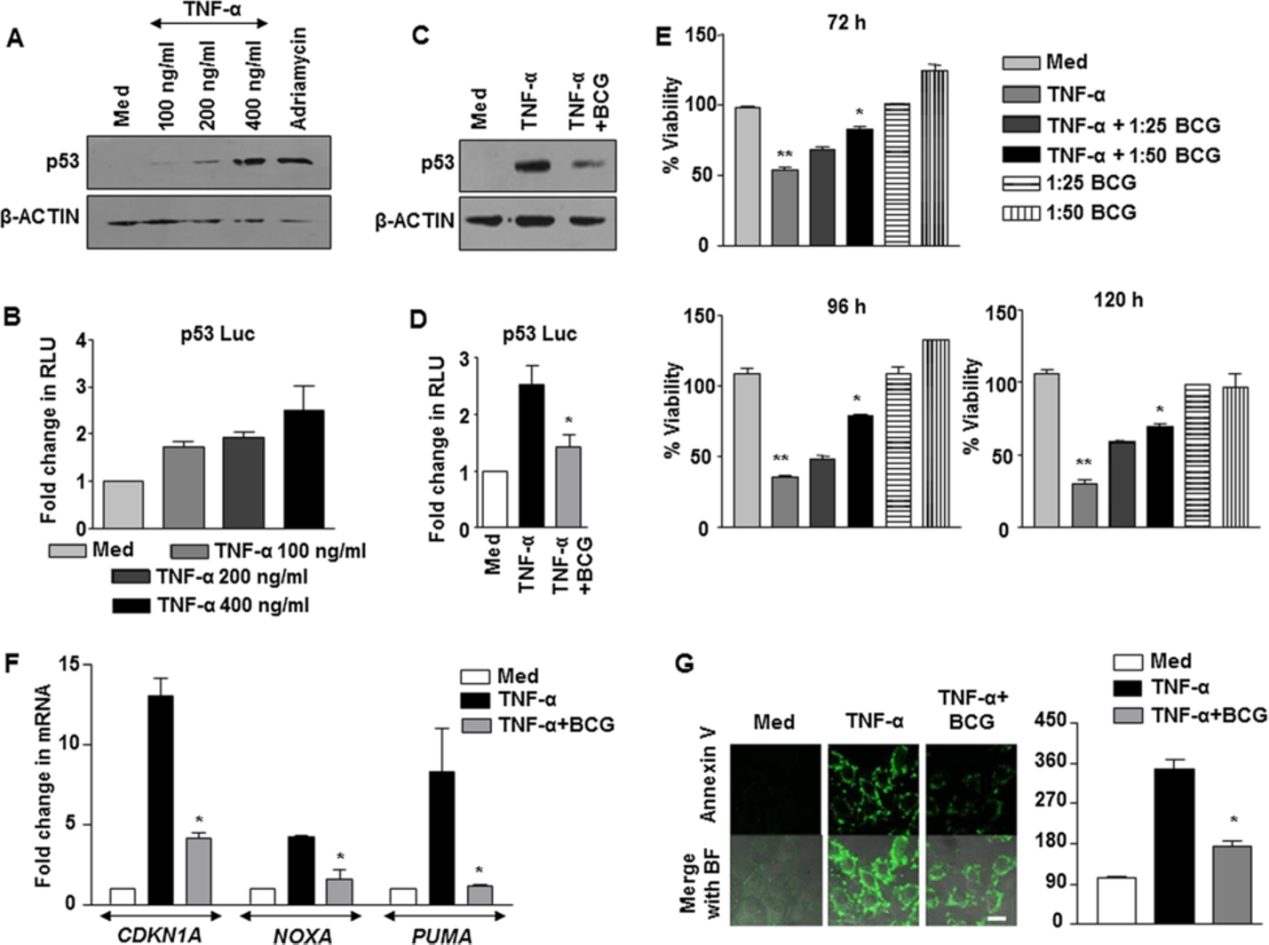
**BCG modulates TNF-α-induced p53. (A)** The expression of p53 in A549 cells treated with various concentration of TNF-α as indicated was analyzed using immunoblotting. Adriamycin (500 ng/ml) was used as positive regulator of p53 expression. **(B)** The p53 promoter luciferase construct was transfected into A549 cells and promoter activity was assayed at various concentrations of TNF-α as indicated. **(C)** A549 cells were infected with BCG for 12 h prior to treatment with TNF-α. p53 expression levels were assessed by immunoblotting. **(D)** A549 cells were transfected with p53 luciferase construct and change in p53 promoter activity with respect to indicated treatments was measured by luciferase assay. **(E)** A549 cells were treated with TNF-α alone or with BCG (MOI of 1: 25 and 1:50, 12 h) and TNF-α as shown. The MTT assay was performed at various time points to measure the cell viability. **(F)** Quantitative real time RT-PCR for p53-responsive pro-apoptotic genes in A549 cells with indicated treatment. **(G)** A549 cells were infected with BCG for 12 h prior to treatment with TNF-α. Representative immunofluorescence images and MFI for Annexin V-FITC staining. Data is representative of mean ± SEM of at least 3 different experiments and all blots are representative of 3 independent experiments. **p < 0.05 (one-way ANOVA), as compared to Med; *p < 0.05 (one-way ANOVA), as compared to TNF-α treated cells. Med, Medium.

To investigate the effect of mycobacterial infection on the tumorigenic properties of alveolar epithelial cells, we performed co-treatment of A549 cells with BCG and TNF-α, a primary cytokine produced during infection [4]. Previous reports suggest that TNF-α induces p53 expression [16] and lung cancers are often associated with deregulated p53 [20]. We thus analyzed the expression of p53 in the current scenario. TNF-α induced p53 expression in A549 cells as assessed by protein levels as well as its promoter activity (Figure 1A and B). Surprisingly, infection of A549 cells with BCG decreased the expression of TNF-α-stimulated p53 (Figure 1C). p53 promoter reporter analysis confirmed the same (Figure 1D). p53 is a tumor suppressor that has been strongly implicated in the process of cell death, especially apoptosis [12]. MTT assays confirmed the ability of BCG to rescue cells from TNF-α-stimulated cell death (Figure 1E). Of note, TNF-α-driven apoptosis is one of the known mechanisms of tumor clearance [21]. Hence, we analyzed the expression of p53-transactivated apoptotic genes such as *CDKN1A/p21*, *NOXA* and *PUMA*. In accordance with the expression of p53, while the responsive genes were induced upon TNF-α treatment, BCG suppressed TNF-α-stimulated expression of *CDKN1A*, *NOXA* and *PUMA* (Figure 1F). Further, as illustrated in Figure 1G and Additional file 1: Figure S1, TNF-α treatment induced apoptosis in lung adenocarcinoma A549 cells. However, BCG-infected cells inhibited TNF-α-arbitrated apoptosis as assessed by Annexin V staining. Together, these results suggest that TNF-α mediates apoptosis via p53 action in A549 cells and BCG surmounts this process.

### BCG induces COP1 via SHH signaling to regulate p53-mediated apoptosis

**Figure 2:**
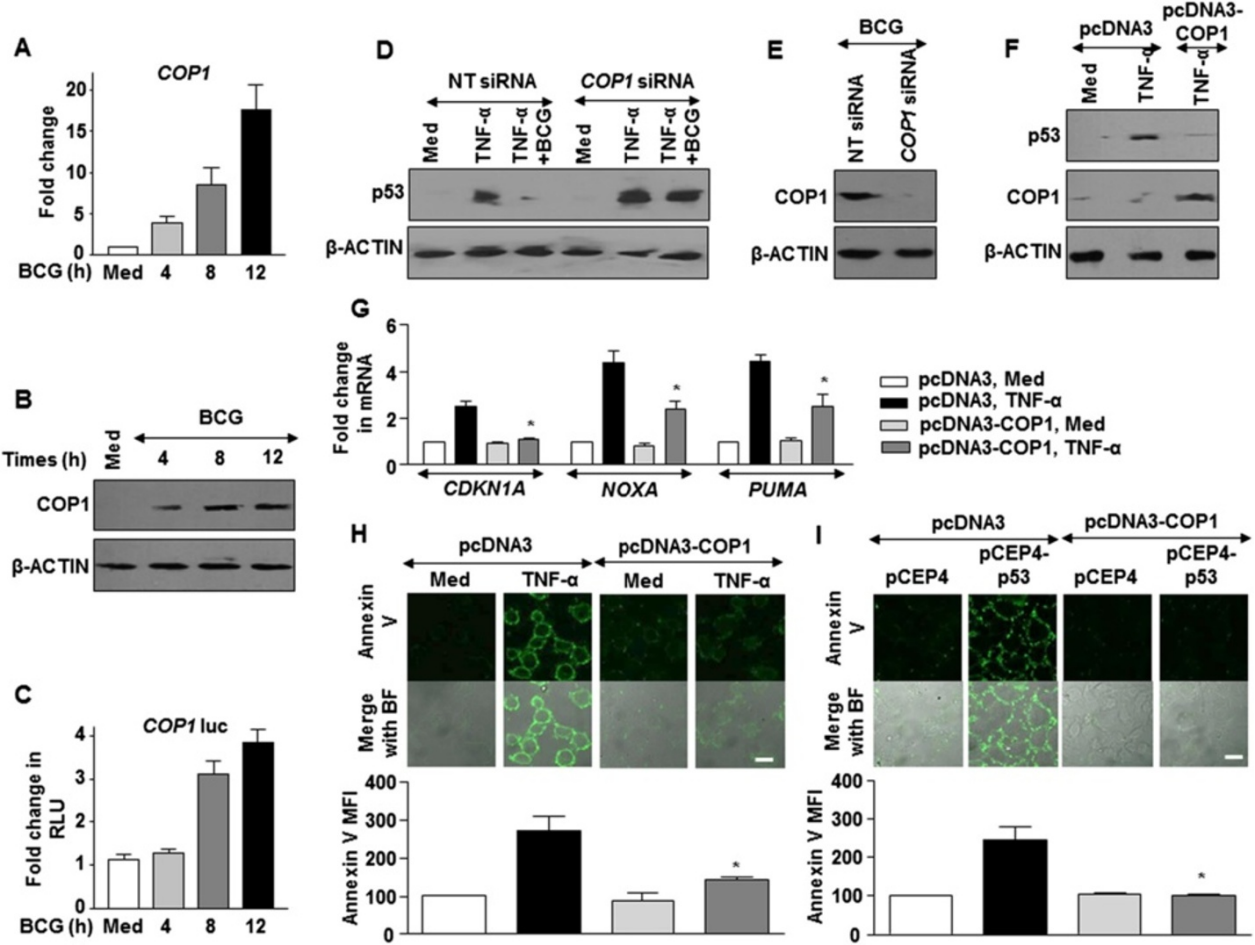
**BCG-induced COP1 regulates p53 expression. (A-C)** A549 cells were infected with BCG at the indicated time points and analyzed for COP1 expression by quantitative real time RT-PCR **(A)** and immunoblotting **(B)**. Alternatively, COP1 promoter activity was measured by luciferase assay **(C)**. **(D and E)** Transient transfection of A549 cells with *COP1* siRNA was performed and treated as indicated. Immunoblotting for p53 **(D)** and validation of *COP1* siRNA by immunoblotting for COP1 **(E)**. **(F and G)** A549 cells were either transfected with vector (pcDNA3) or COP1 overexpression construct (pcDNA3-COP1) and treated with TNF-α as indicated to assess the expression of p53 **(F)** and p53-responsive pro-apoptotic genes **(G)** respectively. **(H and I)** A549 cells were transfected with either COP1 **(H)** or co-transfected with COP1 and p53 **(I)** overexpression constructs and treated as shown. Apoptosis was measured by immunofluorescence microscopy for Annexin V-FITC staining. Panel shows representative immunofluorescence images and quantitation of Annexin V-FITC MFI. Data is representative of mean ± SEM of at least 3 different experiments and all blots are representative of 3 independent experiments. *p < 0.05 (one-way ANOVA), as compared to pcDNA3 transfected, TNF-α treated cells **(G and H)** or pcDNA3 + p53-pCEP4 transfected cells **(I)**. Med, Medium; NT, non-targeting. Bar, 20 μm.

p53 turnover in the cells is often regulated by many E3 ubiquitin ligases including COP1 [15]. Therefore, we examined the expression of COP1 in response to BCG treatment. Notably, treatment of cells with BCG resulted in marked increase of COP1 transcripts as well as the protein (Figure 2A,B and C). Furthermore, while BCG failed to downregulate TNF-α-induced p53 expression in cells transfected with *COP1* siRNA (Figure 2D and E), enforced expression of COP1 significantly reduced the TNF-α-responsive p53 (Figure 2F) and p53-inducible apoptotic genes (Figure 2G) in the cells. Substantiating these results, COP1 overexpression downregulated TNF-α- (Figure 2H) or p53- (Figure 2I) stimulated apoptosis.

**Figure 3:**
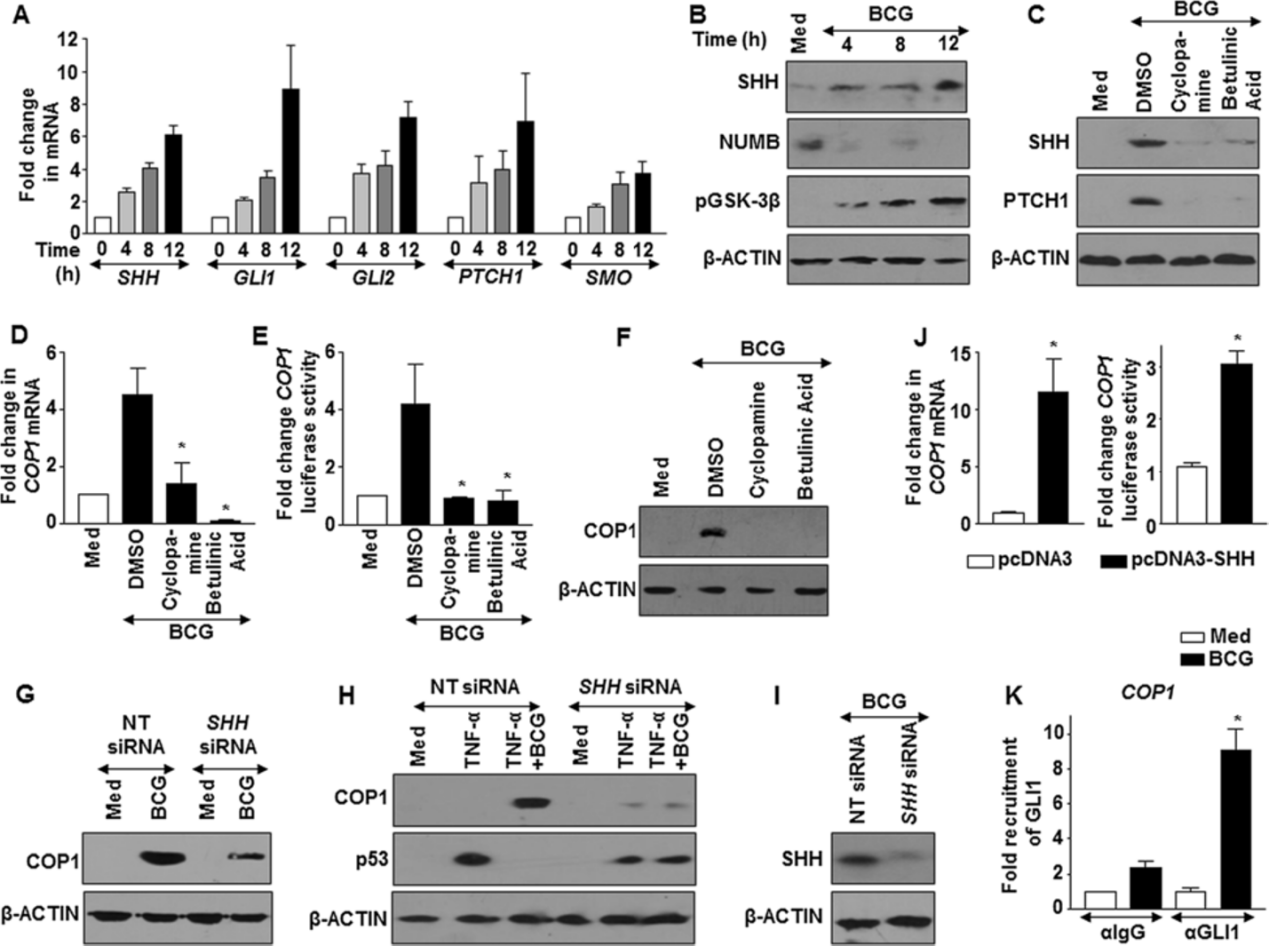
**BCG-mediated SHH signaling induce expression of COP1. (A and B)** The kinetics of SHH signaling activation was assayed by quantitative real time RT-PCR for expression of SHH signaling markers *SHH*, *GLI1*, *GLI2*, *PTCH1* and *SMO* **(A)** and by immunoblotting for SHH, NUMB and pGSK-3β **(B)**. **(C-F)** SHH signaling was inhibited using specific pharmacological inhibitors, Cyclopamine (SMO inhibitor) and Betulinic Acid (GLI inhibitor). Validation of inhibitors used **(C)** and analysis the expression of COP1 by quantitative real time RT-PCR **(D)**, luciferase assay **(E)** and immunoblotting **(F)**. *p < 0.05 (one-way ANOVA), as compared to DMSO treated cells. **(G-I)** A549 cells were transfected with *SHH* specific siRNA to assess COP1 and p53 protein levels after indicated treatment **(G and H)**. Validation of SHH siRNA **(I)**. **(J)** A549 cells overexpressing SHH expression construct were analyzed for COP1 transcripts and COP1 promoter activity. *p < 0.05 (*t*-test), as compared to pcDNA3 transfected cells **(K)** A549 cells were infected with BCG and the recruitment of GLI1 transcription factor at COP1 promoter upon infection with BCG in A549 cells was evaluated by ChIP assay. *p < 0.05 (one-way ANOVA). Data is representative of mean ± SEM, n = 3 and all blots are representative of 3 independent experiments. Med, Medium; NT, non-targeting.

**Figure 4:**
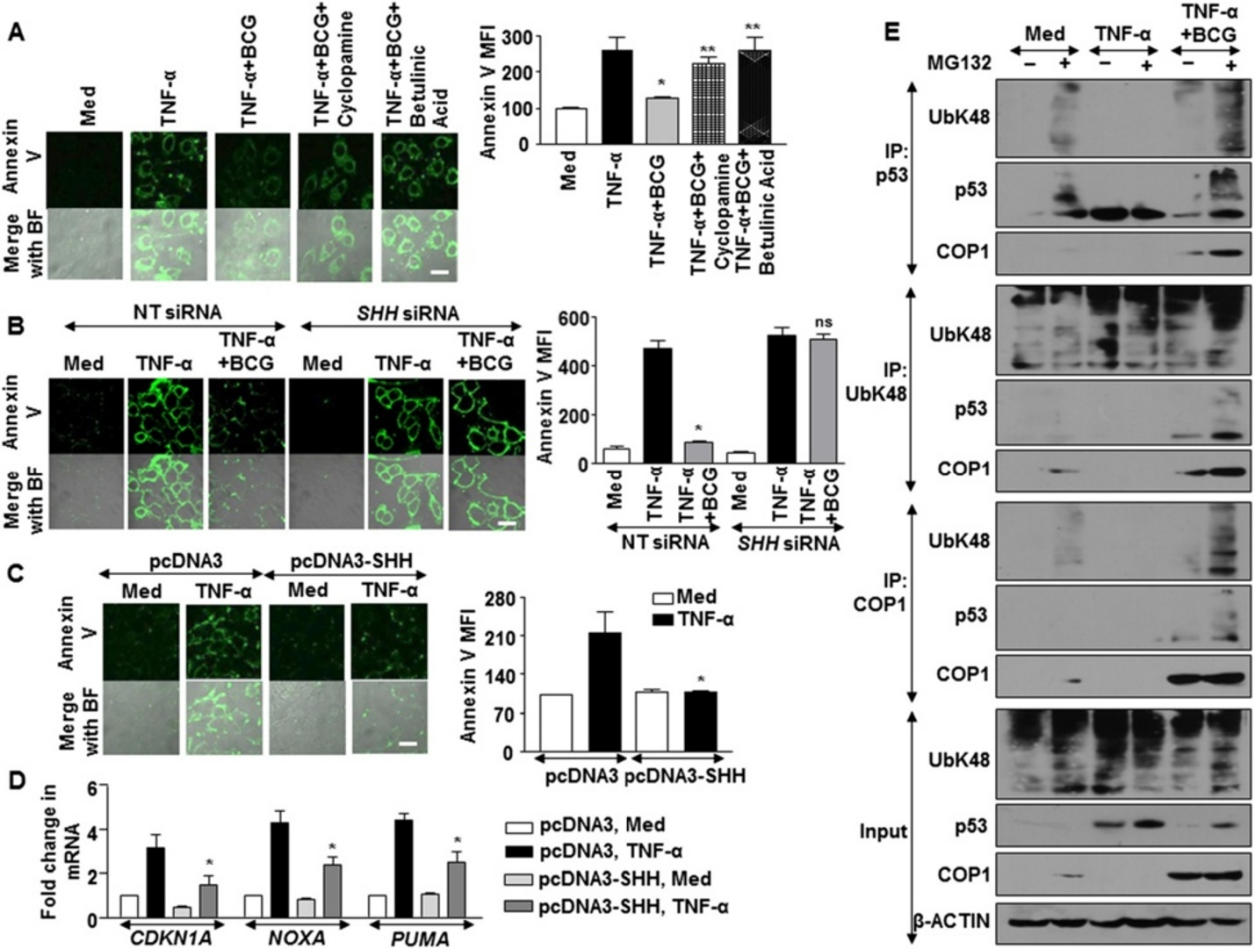
**SHH signaling regulates apoptosis by COP1-mediated p53 degradation. (A-D)** A549 cells were either pretreated with SHH signaling specific inhibitors like Cyclopamine or Betulinic Acid followed by infection with BCG and treatment with TNF-α **(A)** or transfected with *SHH* siRNA followed by BCG and TNF-α treatment **(B)** or transfected with SHH overexpression construct followed by TNF-α treatment **(C and D)**. Analysis of apoptosis in A549 cells determined using Annexin V staining with MFI quantitation **(A-C)** and by quantitative real time RT-PCR for pro-apoptotic gene expressions **(D)**. *p < 0.05 (one-way ANOVA), as compared to TNF-α treated cells **(A)**; or compared to pcDNA3 transfected, TNF-α treated cells **(C)**; **p < 0.005 (one-way ANOVA), as compared to BCG + TNF-α treated cells and ns, not significant, as compared to TNF-α treated *SHH* siRNA-transfected cells. **(E)** A549 cells were infected with BCG for 12 h prior to TNF-α treatment. Cells were then treated with MG132 (20 μM) for 4 h before harvest as indicated. Complexes immunoprecipitated with anti-p53, anti-UbK48 or anti-COP1 were analyzed by immunoblotting with indicated antibodies. Data is representative of mean ± SEM of at least 3 different experiments and all blots are representative of 3 independent experiments. Med, Medium; NT, non-targeting. Bar, 20 μm.

We then explored the molecular mechanisms that promoted BCG-driven COP1 expression in cells. Oncogenic properties of SHH signaling are well established and recent evidences suggested the role of SHH signaling in suppressing p53-mediated apoptosis [22]. Further, BCG was found to activate SHH signaling in macrophages to regulate immune functions [23, 24]. To determine the contribution of SHH signaling in the current study, we first examined the activation of SHH signaling in A549 cells on treatment with BCG. Activation of canonical SHH signaling is assessed by transcript analysis of *SHH*, *GLI1*, *GLI2*, *PTCH1*, *SMO* and protein level expression of SHH, NUMB and GSK-3β. Elevated transcripts, increased SHH protein, pGSK-3β (Ser9) and decreased NUMB indicates active SHH signaling. Utilizing these readouts, BCG was found to induce SHH signaling in A549 cells (Figure 3A and B). Additionally, inhibition of SHH signaling in A549 cells by specific pharmacological inhibitors (Figure 3C,D,E and F) or by RNA interference of *SHH* (Figure 3G,H and I) abrogated BCG-induced COP1 expression and p53 downregulation. Elevated transcripts and promoter activity of COP1 was detected on SHH overexpression (Figure 3J). Importantly, ChIP assay showing recruitment of GLI1 transcription factor to the COP1 promoter in response to BCG treatment indicated that SHH signaling could regulate COP1 expression (Figure 3K). Corroborating these observations, we found that ability of BCG to inhibit TNF-α-stimulated apoptosis was severely compromised in cells that were either pretreated with SHH inhibitors (Figure 4A) or transfected with *SHH* siRNA (Figure 4B). SHH overexpression was sufficient to significantly subdue TNF-α-mediated apoptosis (Figure 4C) and expression of apoptotic genes *CDKN1A*, *NOXA* and *PUMA* (Figure 4D). Further, enhanced K48 ubiquitination of p53 and interaction of COP1 and p53 on BCG pretreatment of A549 cells complement the observations (Figure 4E).

### BCG protects tumors from TNF-α-mediated apoptosis

**Figure 5:**
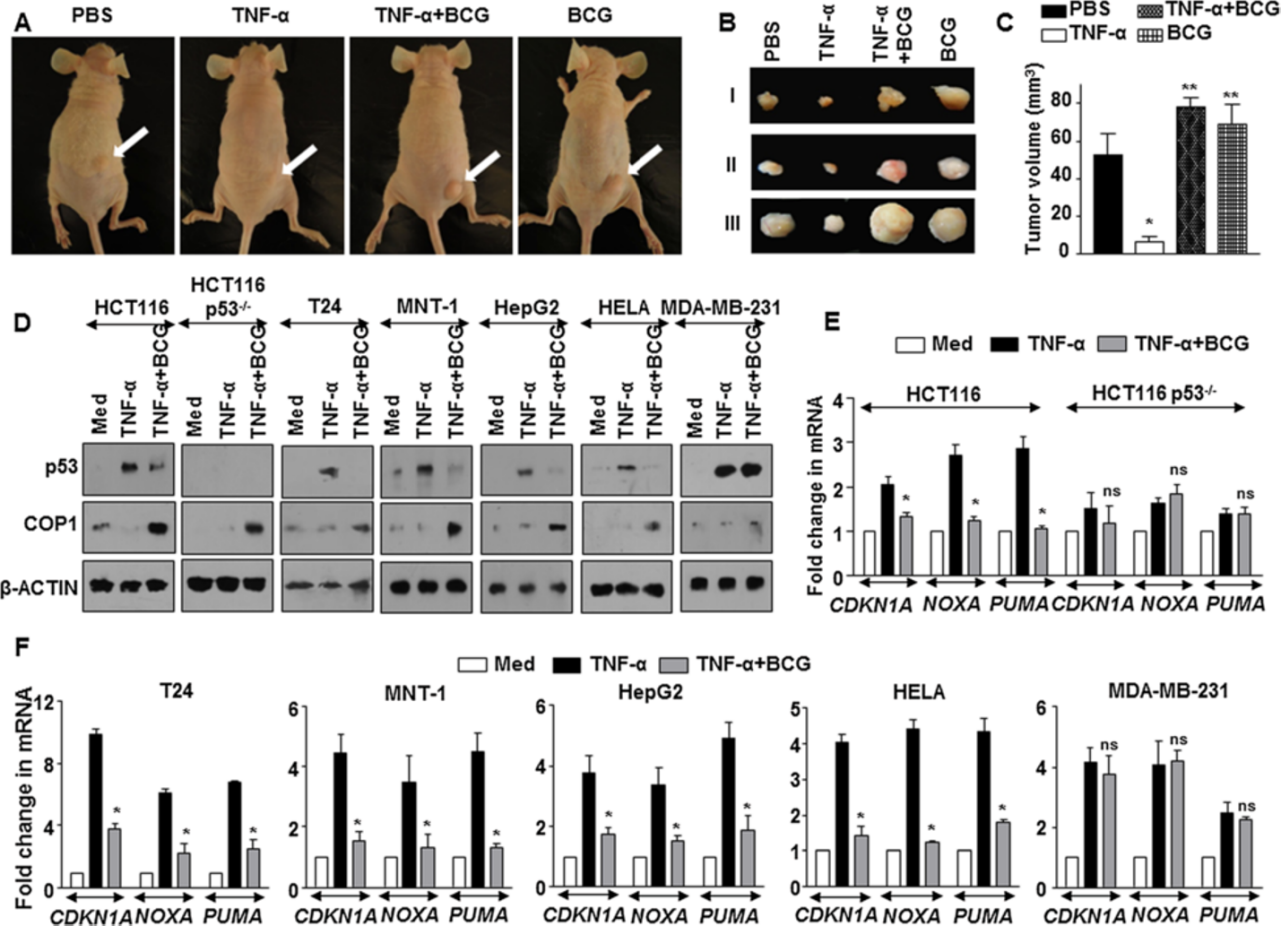
**BCG prevents TNF-α-mediated tumor clearance and inhibits TNF-α-responsive p53 and apoptotic genes. (A-C)** A549 cells were left untreated or treated with TNF-α alone for 12 h or in combination with BCG for 12 h prior to TNF-α treatment. Treated cells (4 × 10^6^) were subcutaneously injected into nude mice. Tumors were harvested after 30 days. **(A)** Representative images of mice with subcutaneous tumors, **(B)** excised tumor and **(C)** tumor volumes. Data is representative of mean ± SEM, *p < 0.05 (one-way ANOVA), as compared to PBS alone tumors and **p < 0.005 (one-way ANOVA), as compared to TNF-α treated tumors. **(D)** The indicated cell lines were infected with BCG for 12 h prior to treatment with TNF-α. p53 and COP1 expression levels were assessed by immunoblotting. **(E and F)** Quantitative real time RT-PCR for p53-responsive pro-apoptotic genes *CDKN1A*, *NOXA* and *PUMA* in different cell lines with indicated treatment. Data is representative of mean ± SEM of at least 3 different experiments and all blots are representative of 3 independent experiments. *p < 0.05 (one-way ANOVA) and ns, not significant, as compared to TNF-α treated cells. Med, Medium.

To evaluate these observations *in vivo*, we chose to analyze the progression of human lung adenocarcinoma A549 xenografts on co-treatment with TNF-α and BCG. Ascertaining the cell line data, while TNF-α-treated xenografts failed to exhibit sustained tumors in nude mice, cells that were infected with BCG prior to TNF-α treatment or infected with BCG alone formed significantly larger tumors (Figure 5A,B and C). This observation was unlike many studies that attributed anti-tumorigenic abilities of BCG instillations [25].

### Inhibition of TNF-α-orchestrated apoptosis by BCG in tested cancers

**Figure 6:**
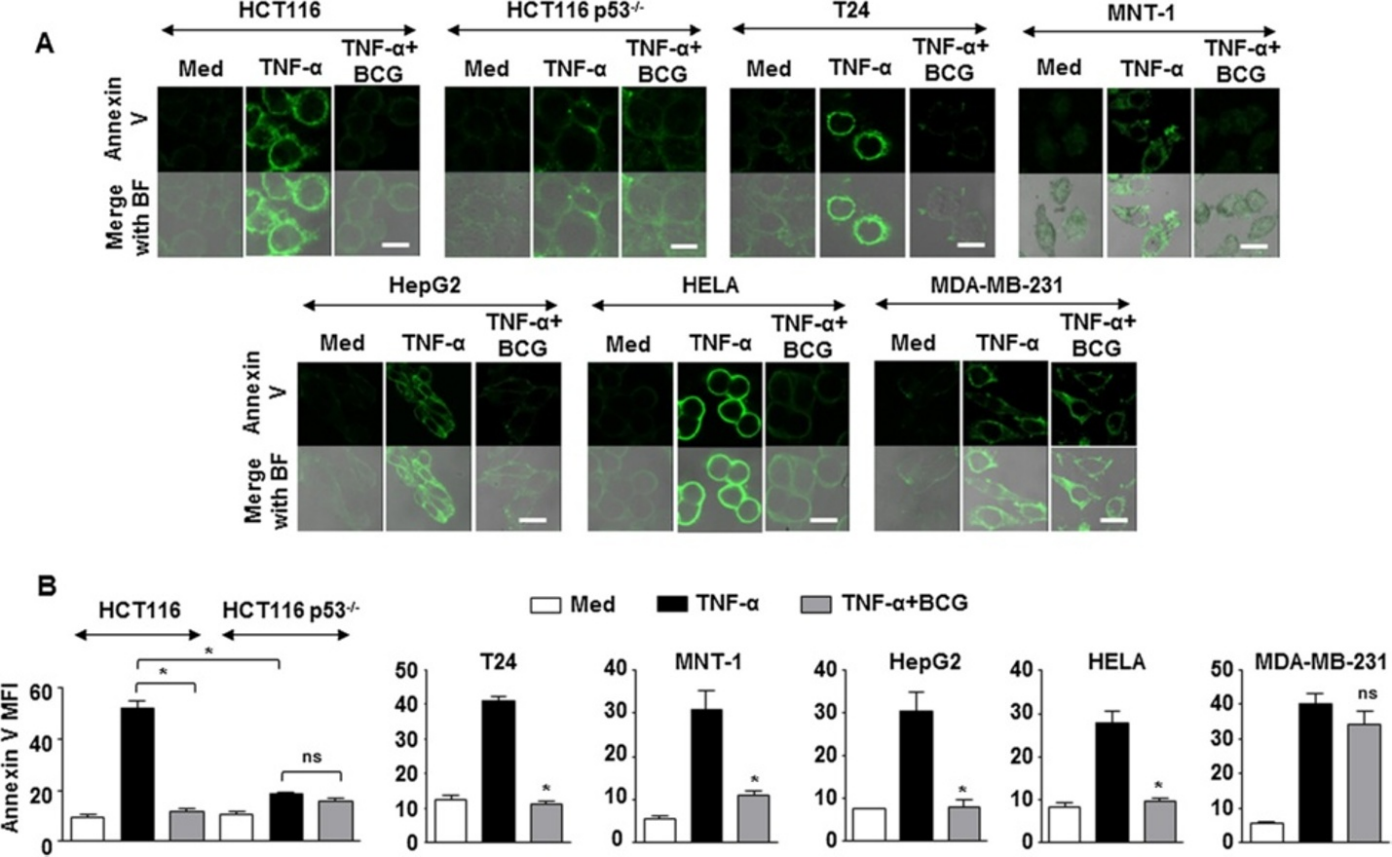
**BCG inhibits TNF-α-mediated apoptosis. (A and B)** HCT116, HCT116 p53^-/-^, T24, MNT-1, HepG2, HELA and MDA-MB-231 cells were infected with BCG for 12 h prior to treatment with TNF-α. Representative immunofluorescence images **(A)** and MFI **(B)** for Annexin V-FITC staining. Data is representative of mean ± SEM of at least 3 different experiments. *p < 0.05 (one-way ANOVA) and ns, not significant, as compared to TNF-α treated cells. Med, Medium. Bar, 20 μm.

BCG is utilized for intravesical immunotherapy for treating cancers such as bladder cancer [26, 27], lung cancer [28, 29], colorectal cancer [30], melanomas [31, 32] hepatocellular carcinoma [33] and cervical cancer [34]. However, large proportion of the patients fails such immunotherapies [27]. Further, TNF-α is also used for cancer therapy [6, 7]. Hence, with the current results, we analyzed the effect of BCG instillation during TNF-α treatment of various tumor cell lines. Corresponding our previous observation, except in HCT116 p53^-/-^ human colorectal carcinoma cells and MDA-MB-231 human breast adenocarcinoma cells, infection of the HCT116 human colorectal carcinoma cells, T24 human urinary bladder carcinoma cells, MNT-1 human melanoma cells, HepG2 human hepatocellular carcinoma cells or HELA human cervical adenocarcinoma cells with BCG decreased the expression of TNF-α-responsive p53 and induced the COP1 expression (Figure 5D). The expression of p53-dependent apoptotic genes such as *CDKN1A*, *NOXA* and *PUMA* followed similar trend (Figure 5E and F). Finally, the ability of BCG to protect the tested tumor cells from TNF-α-mediated apoptosis was also observed in HCT116, T24, MNT-1, HepG2 and HELA cells but not in HCT116 p53^-/-^ cells and MDA-MB-231 cells (Figure 6A and B). In conclusion, these results suggest that inhibition of apoptosis by BCG could fail intravesical immunotherapy.

## Discussion

Interaction of the pathogenic mycobacteria with macrophages and epithelial cells in the alveolar spaces shape the early responses of the infection [1, 2, 35]. Reports suggest that apart from harboring the bacteria, infection-induced cytokines and chemokines secreted by alveolar type II epithelial cells recruit other immune cells such as monocytes, lymphocytes and polymorphonuclear cells [3]. Of note, association of pulmonary tuberculosis with lung cancer has underscored the crucial role for the infected alveolar epithelial cells in maintaining immune and cellular homeostasis during mycobacterial infection [8, 9]. However, the mechanism of infection-induced deregulation is not clear. Here, we found that BCG inhibits the TNF-α-mediated induction of a tumor suppressor, p53 and thus promotes tumor growth.

p53 exhibits a diverse role as a tumor suppressor and hence is called as the guardian of the genome [36]. A number of investigations have emphasized the tumor surveillance property of p53, especially its pro-apoptotic functions [12, 37]. In the current context, BCG targets p53-mediated apoptosis of the cancer cells. Further, COP1 is a RING-finger containing E3-ubiquitin ligase which can maintain the levels of multiple cellular proteins including p53 [15]. Interestingly, reports suggest that COP1 exhibits both oncogenic and tumor suppressive functions depending on the cancer type [38]. Here, we find the oncogenic property of BCG-driven COP1 to promote tumor formation and maintenance by targeting p53.

Multiple signaling pathways are tightly regulated to assert cellular homeostasis and maintenance. Deregulation of such pathways is often implicated in several cancers [39]. Recent studies indicate that aberrant SHH signaling leads to tumorigenesis in several tissues by inducing excessive proliferation and differentiation [18]. In this context, ability of BCG to activate cellular signaling pathways including SHH cascade is well evidenced in macrophages [23, 40, 41]. In the present study, we found that BCG could elevate SHH signaling in A549 cells. Supporting the previous observation [22], the induced SHH signaling inhibits p53-stimulated apoptosis. Notably, COP1 expression is found to be SHH-dependent. COP1 targets p53 for degradation and thus regulates p53-responsive functions.

The history of BCG immunotherapy dates as early as 1976 when Morales and colleagues found its potential to treat superficial bladder cancer [42]. Further, BCG went on to be the first FDA-approved immunotherapy [43]. For a few decades now, BCG immunotherapy is either a standard treatment or constitutes the treatment regime for several cancers. Though the success of such therapies has been tremendous, a significant set of the patients fail BCG therapy. In case of bladder cancer, while 20-30% of patients fail the therapy, 30-50% of them exhibit tumor recurrence [27]. Such cases of failed adjuvant or neoadjuvant BCG therapies and tumor recurrence during various human cancer treatment regimes have initiated alternative or complementary therapies [44]. While several mechanisms of action of BCG immunotherapies have been proposed and tested, we lack clear understanding on the reasons for failed therapies [26]. In the present investigation, we found that the ability of BCG to inhibit apoptosis of cancer cells could be one of the probable reasons that hinder tumor clearance during immunotherapy. Though BCG induces robust inflammatory responses during infection, there are multiple evidences of anti-inflammatory Th2 and regulatory T cell (Treg) functions stimulated by BCG [45, 46]. Interestingly, both Th2 and Treg cells exhibit pro-tumor effects in cancers [47, 48]. Evasion of protective immune responses by BCG could also contribute to failed immunotherapy.

Application of TNF-α as potent cytokine for cancer biotherapy is due to its ability to mediate functions such as apoptosis. To overcome the systemic toxicity associated with such treatments, various strategies including cell-based therapies and gene therapies are under study which aims at site-specific expression of TNF-α [44]. Our study indicates that BCG downregulates TNF-α-arbitrated apoptosis in the tested human cancer cell lines, A549, HCT116, T24, MNT-1, HepG2 and HELA. Interestingly, results in MDA-MB-231 human breast adenocarcinoma cells and HCT116 p53^-/-^ human colorectal carcinoma cells were the outliers. While absence of p53 can be attributed to the reduced TNF-α responses and diminished p53 functions in HCT116 p53^-/-^ cells, the possible reasons for the observations in MDA-MB-231 with mutant p53 is currently under investigation. Further, though BCG treatment, by itself, can induce the expression of TNF-α in immune cells [23] and during BCG therapy [49], in the current study we addressed the ability of BCG to revert the TNF-α responses such as apoptosis during therapy. However, BCG failed to protect A549 cells against apoptosis induced by other death ligands of the TNF family such as TRAIL (Additional file 2: Figure S2). This observation is in line with the available literature that suggests that TRAIL induces apoptosis regardless of the p53 status in the cells [50]. Further, the specificity of the response was also assessed in macrophages, the main host of *Mycobacterium*. BCG infection of PMA-stimulated THP-1 macrophages failed to protect the cells from TNF-α-mediated apoptosis (Additional file 3: Figure S3). This observation is in line with existing literatures that suggest that BCG induces apoptosis of the infected macrophages [51–53]. However, TNF-α-induced p53 expression was slightly downregulated during BCG infection of THP-1 cells. This suggests that there could be p53-independent mechanisms that BCG adopts for inducing apoptosis in macrophages [53]. Interestingly, treatment of A549 cells with BCG lysate, Pam3CSK4 (a TLR2/1 agonist), LPS (a TLR4 agonist) or R848 (a TLR7/8 agonist) showed induced COP1 expression and corresponding inhibition of TNF-α-mediated p53 expression (Additional file 4: Figure S4A). However, except for LPS treatment, induction of apoptosis by TNF-α was downregulated by BCG lysate, Pam3CSK4 and R848 (Additional file 4: Figure S4B and C). This suggests that stimulation of TLR1/2/7/8 could lead to suppression of TNF-α-mediated responses in A549 cells.

## Conclusion

**Figure 7:**
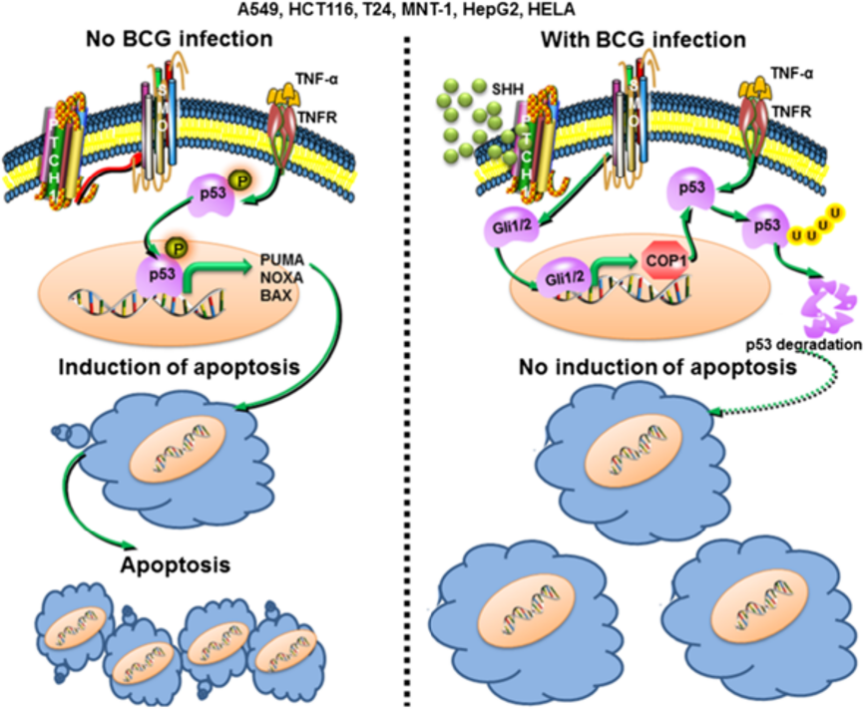
**Model.** TNF-α regulates the progression of cancers by promoting p53-dependent apoptosis. However, the presence of BCG induces SHH signaling-dependent expression of COP1, a regulator of p53 activity. COP1 targets p53 for ubiquitin-mediated proteasomal degradation. These events culminate into inhibition of apoptosis and promotion of tumorigenicity.

In summary, we show that BCG suppressed the apoptotic functions of TNF-α in many cancer cell lines thereby promoting tumorigenesis. Here, SHH-responsive E3 ligase COP1 aids in downregulating the TNF-α-driven p53 expression and functions (Figure 7). Our results suggest that inhibition of apoptosis by *M. bovis* BCG could result in unsuccessful intravesical immunotherapy. Thus the study underscores the need for alternate agents for immunotherapy.

## Methods

### Cell-lines and bacteria

A549 human lung adenocarcinoma, HCT116, HCT116 p53^-/-^ human colorectal carcinoma, HELA human cervical adenocarcinoma cell lines and THP-1 human monocytic cells were cultured in DMEM (Gibco-Life Technologies, Carlsbad, CA, USA) containing 10% FBS (Gibco-Life Technologies). T24 human urinary bladder carcinoma cells were cultured in McCoy’s 5A modified medium (Sigma-Aldrich, St. Louis, MO, USA). MNT-1 human melanoma cell line was maintained in MNT-1 media containing DMEM supplemented with 10% AIMV medium, 15% FBS, 1% glutamine, 1% sodium pyruvate and 1% non-essential amino acids (all from Gibco-Life Technologies). HepG2 human hepatocellular carcinoma cell line was cultured in EMEM (Sigma-Aldrich) containing 10% FBS. *M. bovis* BCG Pasteur 1173P2 was obtained from Pasteur Institute, Paris, France. BCG-RFP was generated in the lab. Bacteria were grown to mid-log phase and used at 10 multiplicity of infection (MOI) in all the experiments.

### Reagents and antibodies

General laboratory chemicals were obtained from Sigma-Aldrich or Merck Millipore (Darmstadt, Germany). TNF-α and TRAIL were purchased from PeproTech Inc (Rocky Hill, NJ, USA). Pam3CSK4, LPS and R848 were purchased from ImmunoTools (Friesoythe, Germany). Anti-β-ACTIN antibody was bought from Sigma-Aldrich. Anti-p53 and anti-COP1 antibodies were obtained from Santa Cruz Biotechnology Ltd (Dallas, Texas, USA). Anti-SHH, anti-GLI1, anti-PTCH1, anti-NUMB, anti-Ser9 phospho GSK-3β and anti-UbK48 were purchased from Cell Signaling Technology (Danvers, MA, USA). Annexin V- fluorescein isothiocyanate (FITC) was from Miltenyi Biotech (Bergisch Gladbach, Germany). HRP conjugated anti-rabbit IgG and anti-mouse IgG were obtained from Jackson ImmunoResearch (West Grove, PA, USA).

### Xenografts in nude mice

Nude mice (nu/nu) were maintained in the Central Animal Facility, Indian Institute of Science. All studies involving mice were carried out after the approval from the Institutional Ethics Committee for Animal Experimentation as well as from Institutional Biosafety Committee. Subcutaneous injection of mice with 4 × 10^6^ A549 cells into its right flank was carried out. The xenografts were monitored regularly for a period of 30 days and tumor sizes were measured with a caliper. After 30 days, tumors were excised and the tumor volume was measured by the formulae V = (LxW^2^) × 0.52, where V is volume, L is length, W is width.

### Treatment with pharmacological reagents

In the experiments utilizing inhibitors, cells were treated with the given inhibitor for 1 h before the experimental treatments at following concentrations: Cyclopamine (10 μM), Betulinic Acid (10 μM) (both from Calbiochem-Merck Millipore, Darmstadt, Germany). DMSO at 0.1% concentration was used as the vehicle control. In all experiments involving pharmacological reagents, a tested concentration was used after careful titration experiments assessing the viability of the cells using the MTT (3-(4,5-Dimethylthiazol-2-yl)-2,5-diphenyltetrazolium bromide) assay.

### MTT assay

Survival of the A549 cells were evaluated by MTT assay as described previously [54]. Briefly, a cell density of 1.5 × 10^3^/well was seeded in a 96-well plate and the experiment was carried out. 20 μl of 5 mg/ml MTT reagent was added to the wells 3 h prior to the completion of the experiment. The formazan crystals formed in the living cells were dissolved in 200 μl of DMSO which was added to the washed cells. Absorbance of the solution was measured at 550 nm using an ELISA reader (Molecular Devices, Sunnyvale, CA, USA).

### Transfection studies

A549 cells were transiently transfected with 5 μg of COP1, p53, SHH or β-galactosidase overexpression constructs or COP1, p53 luciferase constructs using low m.w. polyethylenimine (Sigma-Aldrich). In case of experiments involving siRNA, A549 cells were transfected with 100 nM siRNA. *SHH*, *GLI1*, *COP1*, non-targeting siRNA and siGLO Lamin A/C were obtained from Dharmacon, Inc. (Lafayette, CO, USA) as siGENOME™ SMARTpool reagents, which contain a pool of four different double-stranded RNA oligonucleotides. Transfection efficiency was found to be more than 70% in all the experiments as determined by counting the number of siGLO Lamin A/C positive cells. In all cases, 48 h post-transfection, the cells were treated as indicated and processed for analysis.

### Immunofluorescence

Cells were seeded on to coverslips and treated as indicated. Annexin V-FITC staining was carried out according to manufacturer’s instructions. The cells were fixed with 3.7% paraformaldehyde for 15 min at room temperature and the coverslips were mounted on a slide with glycerol. Confocal images were taken on Zeiss LSM 710 and Zeiss LSM 510 Meta confocal laser scanning microscopes (Carl Zeiss AG, Oberkochen, Germany) using a plan-Apochromat 63X/1.4 Oil DIC objective and images were analyzed using ZEN 2009 software (Carl Zeiss AG).

### RNA isolation and quantitative real time RT-PCR

Total RNA from experimentally treated cell lines was isolated using TRI reagent (Sigma-Aldrich). 2 μg of total RNA was converted into cDNA using First strand cDNA synthesis kit (Bioline, London, UK). Quantitative real time RT-PCR for quantification of the target gene expression was performed using SYBR Green PCR mixture (KAPA Biosystems, Woburn, MA, USA). All the experiments were repeated at least three times independently to ensure the reproducibility of the results. *GAPDH* was used as internal control. Additional file 5: Table S1 contains the primers used for quantitative real time RT-PCR.

### Immunoblotting

RIPA buffer [50 mM Tris–HCl (pH 7.4), 1% NP-40, 0.25% Sodium deoxycholate, 150 mM NaCl, 1 mM EDTA, 1 mM PMSF, 1 μg/ml of each aprotinin, leupeptin, pepstatin, 1 mM Na_3_VO_4_ and 1 mM NaF] was used to obtain the total cell lysates. Protein in each sample was estimated using Bradford assay; equal amount of protein was resolved on a 12% SDS-polyacrylamide gel and transferred to polyvinylidene difluoride (PVDF) membranes (Millipore) by the semi-dry transfer (Bio-Rad, Hercules, CA, USA) method. Blocking with 5% nonfat dry milk powder in TBST [20 mM Tris–HCl (pH 7.4), 137 mM NaCl, and 0.1% Tween 20] for 60 min was done to prevent nonspecific binding. The blots were incubated overnight at 4°C with primary antibody followed by incubation with anti-rabbit-HRP or anti-mouse-HRP secondary antibody in 5% BSA for 2 h. The immunoblots were developed with enhanced chemiluminescence detection system (PerkinElmer, Waltham, MA, USA) as per manufacturer’s instructions. β-ACTIN was used as loading control. Blots were incubated in the stripping buffer [62.5 mM Tris–HCl (pH 6.8), 2% SDS and 0.7% β-mercaptoethanol] at 60°C on a shaker for probing another protein in the same region of the PVDF membrane.

### Luciferase assays

Transfected cells were lysed in Reporter lysis buffer (Promega, Madison, WI, USA) and assayed for luciferase activity using Luciferase Assay Reagent (Promega) as per the manufacturer’s instructions. β-galactosidase activity utilizing O-nitrophenol β-D-galactopyranoside (HiMedia Laboratories Pvt Ltd, Mumbai, Maharashtra, India) was performed to normalize the transfection efficiencies.

### Chromatin immunoprecipitation assay

Chromatin immunoprecipitation (ChIP) assay was carried out using a protocol provided by Upstate Biotechnology, Inc. (Lake Placid, NY, USA), with certain modifications. Briefly, cells were fixed with 1.42% formaldehyde for 15 min at room temperature followed by inactivation of formaldehyde using 125 mM glycine. Modified RIPA buffer containing 1% Triton X-100 was used for cell lysis and samples were sonicated to obtain sheared chromatin fragments of 500 bp. Anti-GLI1 or rabbit preimmune sera immunoprecipitated DNA was purified and analyzed by quantitative PCR. All results were normalized to amplification of 28S rRNA. ChIP experiments were repeated at least three times and the primers utilized are listed in Additional file 5: Table S1.

### Immunoprecipitation assay

Immunoprecipitation (IP) assays were carried out using protocol provided by Millipore with certain modifications. Briefly, the cells were gently lysed in ice-cold RIPA buffer on an orbital shaker. The cell lysates were incubated with anti-UbK48, anti-p53, anti-COP1 or rabbit preimmune sera at 4°C for 2 h on an orbital shaker. The immunocomplexes were captured using Protein A (Bangalore Genei) agarose at 4°C for 2 h. The beads were harvested, washed, and boiled in 5× Laemmli buffer for 10 min. The samples were separated by SDS-PAGE and further subjected for immunoblotting.

### Statistical analysis

Levels of significance for comparison between samples were determined by the Student *t* test distribution and one-way ANOVA. The data in the graphs are expressed as the mean ± SEM for 5 or 6 values from 3 independent experiments and p values < 0.05 were defined as significant. GraphPad Prism 5.0 software (GraphPad Software, Inc., San Diego, CA, USA) was used for all the statistical analysis.

## Electronic supplementary material

Additional file 1: Figure S1: BCG inhibits TNF-α-induced apoptosis. (A) A549 cells were infected with BCG-RFP for 12 h prior to TNF-α treatment. Representative immunofluorescence images for Annexin V-FITC staining and BCG-RFP. Data is representative of 3 different experiments. Med, Medium. Bar, 5 μm. (DOC 2 MB)

Additional file 2: Figure S2: BCG failed to downregulate TRAIL-induced apoptosis. (A) A549 cells were infected with BCG for 12 h prior to treatment with 20 ng/ml TRAIL. Representative immunofluorescence images and MFI for Annexin V-FITC staining. Data is representative of mean ± SEM of at least 3 different experiments. ns, not significant, as compared to TNF-α treated cells. Med, Medium. Bar, 20 μm. (DOC 1 MB)

Additional file 3: Figure S3: BCG failed to downregulate TNF-α-induced apoptosis in macophages. (A and B) PMA-stimulated THP-1 cells were infected with BCG for 12 h prior to TNF-α treatment. Expression of p53 and COP1 were assessed by immunoblotting with total cell lysate (A) and representative immunofluorescence images and MFI for Annexin V-FITC staining (B). Data is representative of mean ± SEM of at least 3 different experiments and all blots are representative of 3 independent experiments. ns, not significant, as compared to TNF-α treated cells. Med, Medium. Bar, 20 μm. (DOC 758 KB)

Additional file 4: Figure S4: Ability of various innate receptor agonists to inhibit TNF-α-induced apoptosis. (A-C) A549 cells were either infected with BCG or stimulated with BCG lysate, Pam3CSK4 (1 μg/ml), LPS (50 ng/ml) or R848 (1 μg/ml) for 12 h prior to treatment with TNF-α. Immunoblotting analysis of p53 and COP1 (A) and MFI (B) and representative immunofluorescence images (C) for Annexin V-FITC staining. Data is representative of mean ± SEM of at least 3 different experiments and all blots are representative of 3 independent experiments. *p < 0.05 (one-way ANOVA) and ns, not significant, as compared to TNF-α treated cells. Med, Medium. Bar, 20 μm. (DOC 2 MB)

Additional file 5: Table S1: Primers used in the study. (DOC 44 KB)

## Abbreviations

BCG: *M. bovis* Bacillus Calmette-Guérin
ChIP: Chromatin immunoprecipitation
COP1: Constitutively photomorphogenic 1
IP: Immunoprecipitation
MTT: 3-(4,5-Dimethylthiazol-2-yl)-2,5-diphenyltetrazolium bromide
PTCH1: Patched-1
SMO: Smoothened
SHH: Sonic hedgehog
Treg: Regulatory T cell
TNF: Tumor necrosis factor.
