# Supplementary material for: Mycobacterium bovis BCG promotes tumor cell survival from tumor necrosis factor-α-induced apoptosis
Source: Mol Cancer. 2014 Sep 11;13:210. doi: 10.1186/1476-4598-13-210 (PMC4174669; doi:10.1186/1476-4598-13-210)
Supplement: Supplementary file 1 — Additional file 1: Figure S1: BCG inhibits TNF-α-induced apoptosis. (A) A549 cells were infected with BCG-RFP for 12 h prior to TNF-α treatment. Representative immunofluorescence images for Annexin V-FITC staining and BCG-RFP. Data is representative of 3 different experiments. Med, Medium. Bar, 5 μm. (DOC 2 MB) [file 12943_2014_1415_MOESM1_ESM.doc]

**Additional file 1: Figure S1**

**
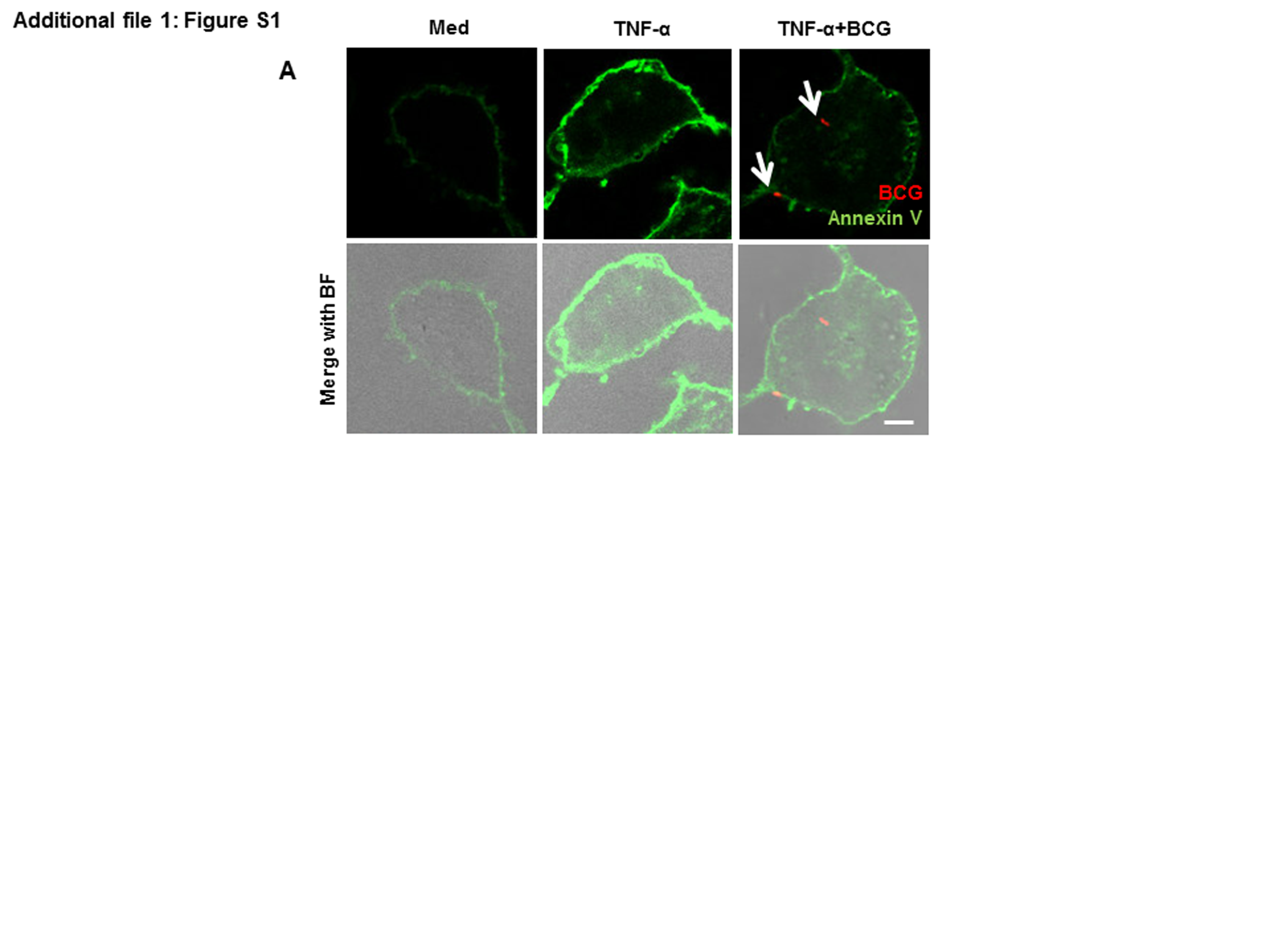
**

**Figure S1. BCG inhibits TNF-α-induced apoptosis. (A)** A549 cells were infected with BCG-RFP for 12 h prior to TNF-α treatment. Representative immunofluorescence images for Annexin V-FITC staining and BCG-RFP. Data is representative of 3 different experiments. Med, Medium. Bar, 5 µm.
