# Supplementary material for: Mycobacterium bovis BCG promotes tumor cell survival from tumor necrosis factor-α-induced apoptosis
Source: Mol Cancer. 2014 Sep 11;13:210. doi: 10.1186/1476-4598-13-210 (PMC4174669; doi:10.1186/1476-4598-13-210)
Supplement: Supplementary file 2 — Additional file 2: Figure S2: BCG failed to downregulate TRAIL-induced apoptosis. (A) A549 cells were infected with BCG for 12 h prior to treatment with 20 ng/ml TRAIL. Representative immunofluorescence images and MFI for Annexin V-FITC staining. Data is representative of mean ± SEM of at least 3 different experiments. ns, not significant, as compared to TNF-α treated cells. Med, Medium. Bar, 20 μm. (DOC 1 MB) [file 12943_2014_1415_MOESM2_ESM.doc]

**Additional file 2: Figure S2**

**
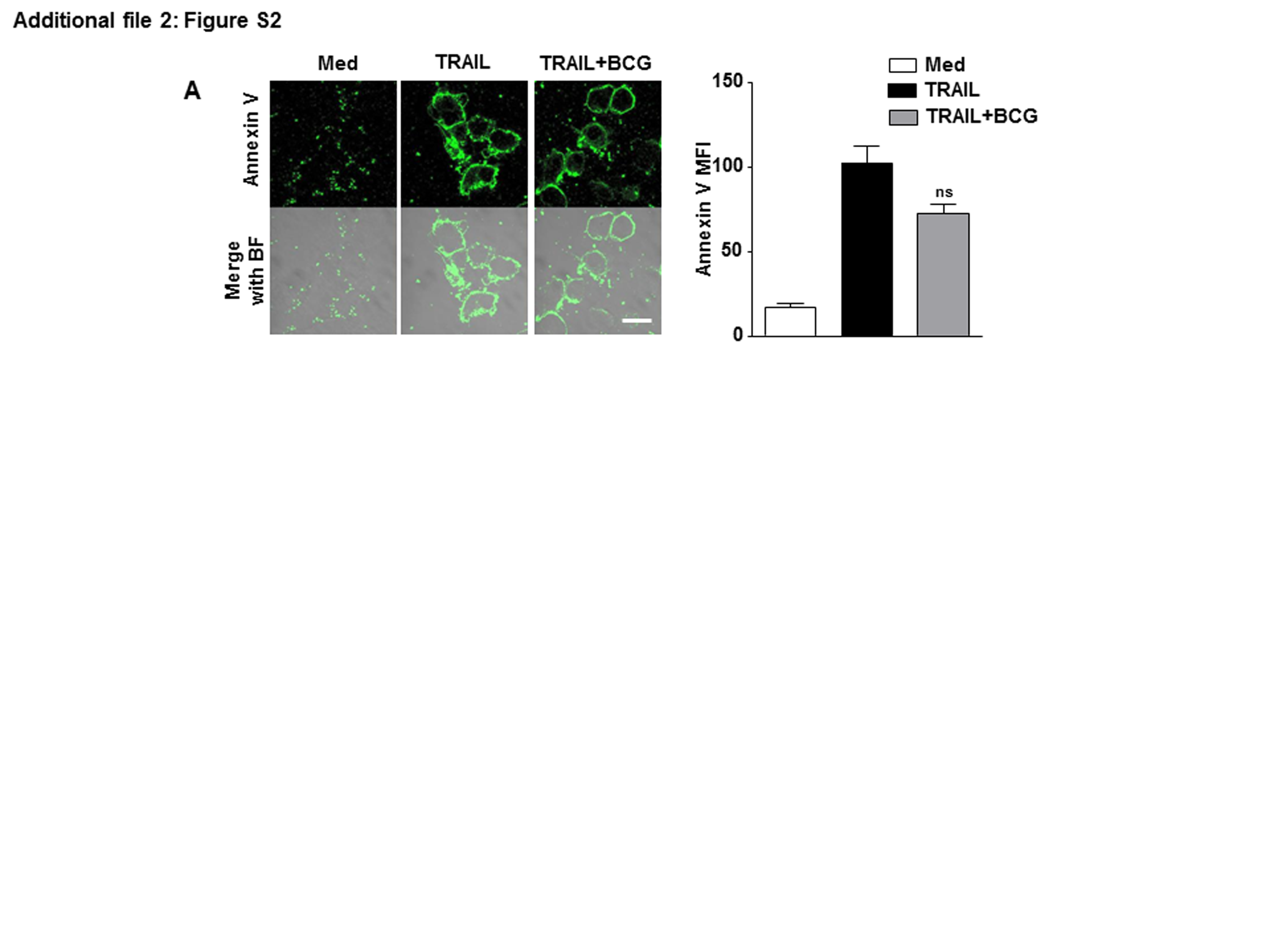
**

**Figure S2. BCG failed to downregulate TRAIL-induced apoptosis. (A)** A549 cells were infected with BCG for 12 h prior to treatment with 20 ng/ml TRAIL. Representative immunofluorescence images and MFI for Annexin V-FITC staining. Data is representative of mean ± SEM of at least 3 different experiments. ns, not significant, as compared to TNF-α treated cells. Med, Medium. Bar, 20 µm.
