# Supplementary material for: Mycobacterium bovis BCG promotes tumor cell survival from tumor necrosis factor-α-induced apoptosis
Source: Mol Cancer. 2014 Sep 11;13:210. doi: 10.1186/1476-4598-13-210 (PMC4174669; doi:10.1186/1476-4598-13-210)
Supplement: Supplementary file 3 — Additional file 3: Figure S3: BCG failed to downregulate TNF-α-induced apoptosis in macophages. (A and B) PMA-stimulated THP-1 cells were infected with BCG for 12 h prior to TNF-α treatment. Expression of p53 and COP1 were assessed by immunoblotting with total cell lysate (A) and representative immunofluorescence images and MFI for Annexin V-FITC staining (B). Data is representative of mean ± SEM of at least 3 different experiments and all blots are representative of 3 independent experiments. ns, not significant, as compared to TNF-α treated cells. Med, Medium. Bar, 20 μm. (DOC 758 KB) [file 12943_2014_1415_MOESM3_ESM.doc]

**Additional file 3: Figure S3**

**
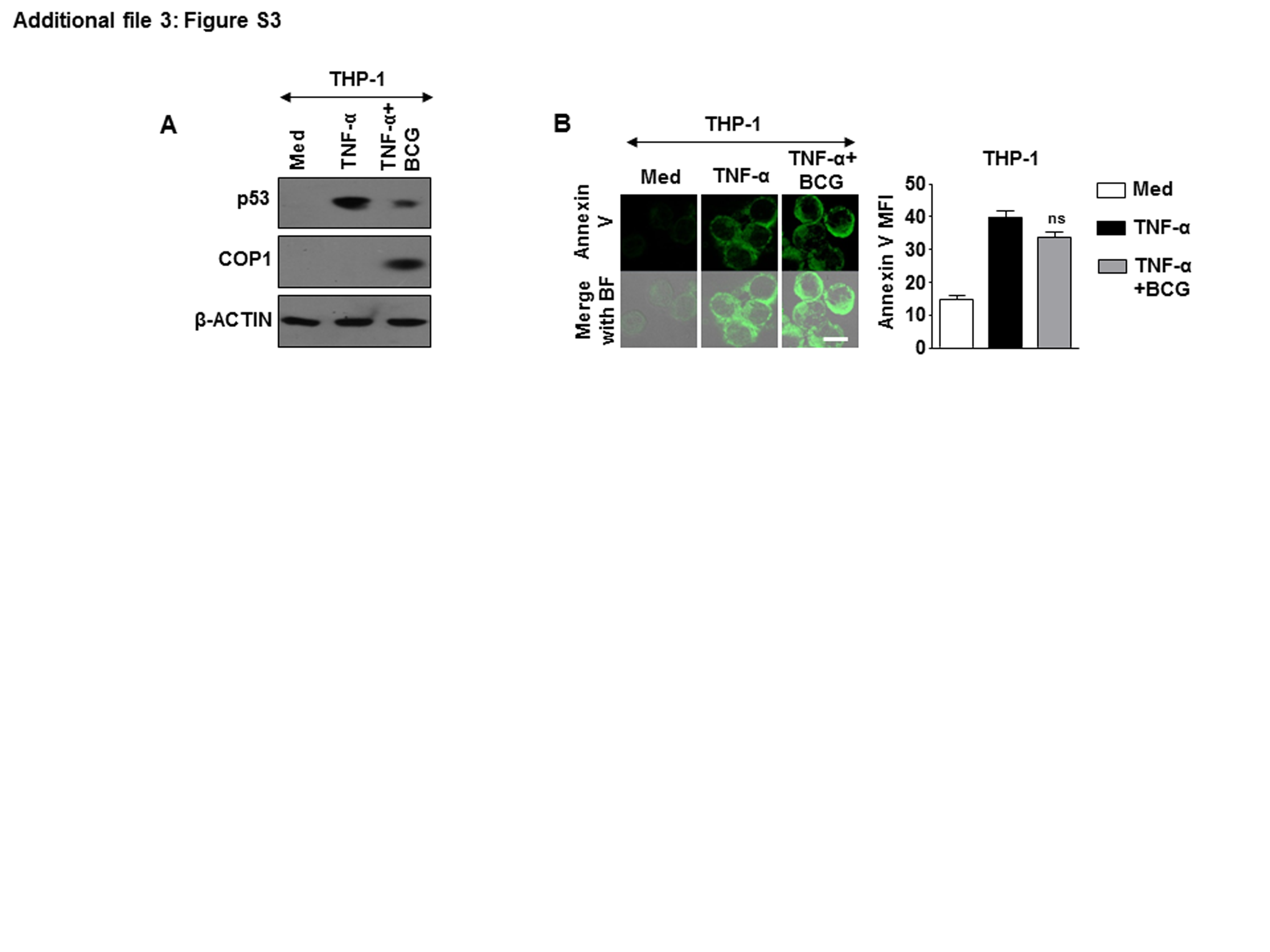
**

**Figure S3. BCG failed to downregulate TNF-α-induced apoptosis in macophages. (A and B)** PMA-stimulated THP-1 cells were infected with BCG for 12 h prior to TNF-α treatment. Expression of p53 and COP1 were assessed by immunoblotting with total cell lysate **(A)** and representative immunofluorescence images and MFI for Annexin V-FITC staining **(B)**. Data is representative of mean ± SEM of at least 3 different experiments and all blots are representative of 3 independent experiments. ns, not significant, as compared to TNF-α treated cells. Med, Medium. Bar, 20 µm.
