# Supplementary material for: Mycobacterium bovis BCG promotes tumor cell survival from tumor necrosis factor-α-induced apoptosis
Source: Mol Cancer. 2014 Sep 11;13:210. doi: 10.1186/1476-4598-13-210 (PMC4174669; doi:10.1186/1476-4598-13-210)
Supplement: Supplementary file 4 — Additional file 4: Figure S4: Ability of various innate receptor agonists to inhibit TNF-α-induced apoptosis. (A-C) A549 cells were either infected with BCG or stimulated with BCG lysate, Pam3CSK4 (1 μg/ml), LPS (50 ng/ml) or R848 (1 μg/ml) for 12 h prior to treatment with TNF-α. Immunoblotting analysis of p53 and COP1 (A) and MFI (B) and representative immunofluorescence images (C) for Annexin V-FITC staining. Data is representative of mean ± SEM of at least 3 different experiments and all blots are representative of 3 independent experiments. *p < 0.05 (one-way ANOVA) and ns, not significant, as compared to TNF-α treated cells. Med, Medium. Bar, 20 μm. (DOC 2 MB) [file 12943_2014_1415_MOESM4_ESM.doc]

**Additional file 4: Figure S4**

**
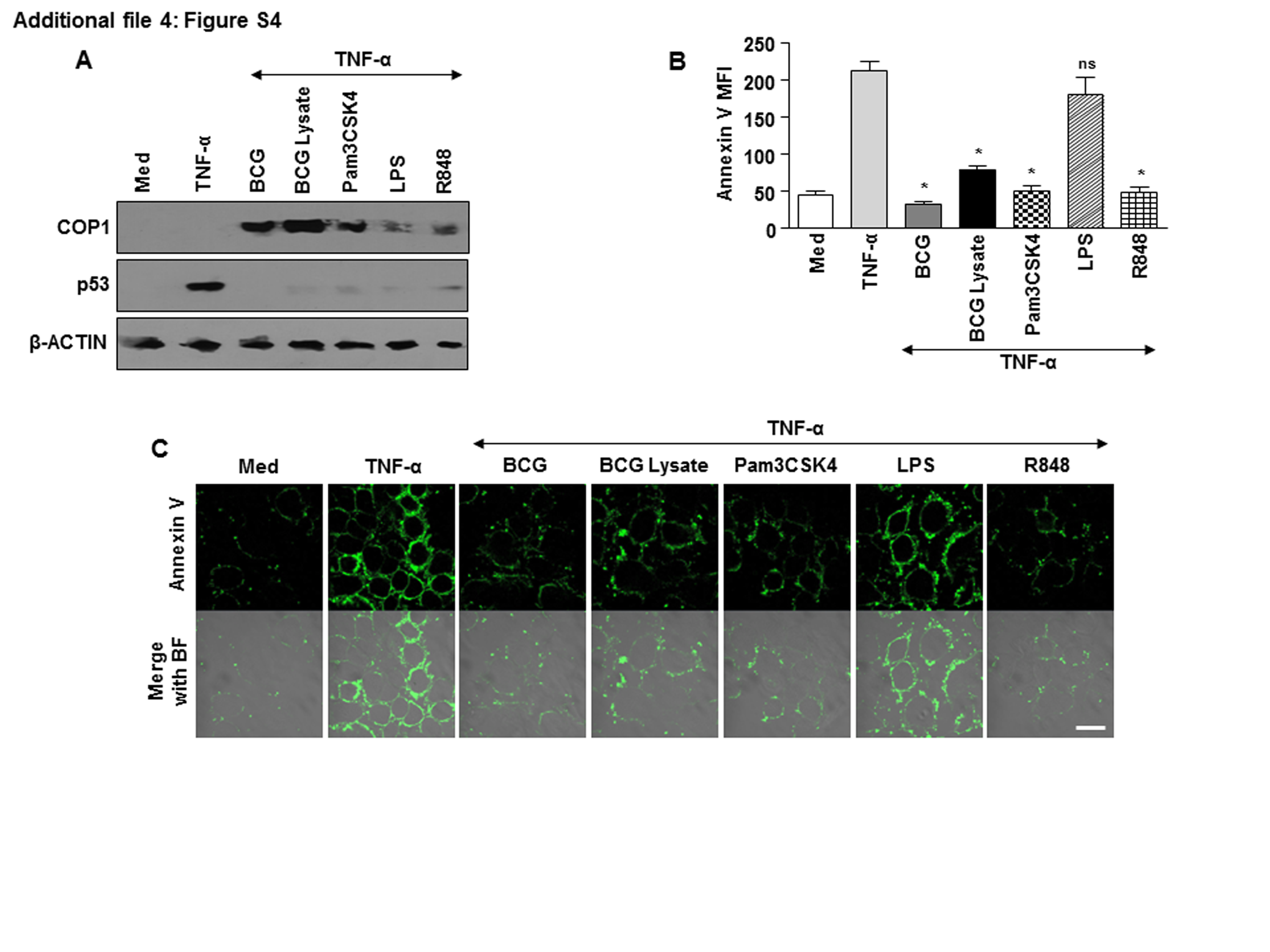
**

**Figure S4. Ability of various innate receptor agonists to inhibit TNF-α-induced apoptosis. (A-C)** A549 cells were either infected with BCG or stimulated with BCG lysate, Pam3CSK4 (1 µg/ml), LPS (50 ng/ml) or R848 (1 µg/ml) for 12 h prior to treatment with TNF-α. Immunoblotting analysis of p53 and COP1 **(A)** and MFI **(B)** and representative immunofluorescence images **(C)** for Annexin V-FITC staining. Data is representative of mean ± SEM of at least 3 different experiments and all blots are representative of 3 independent experiments. * p<0.05 (one-way ANOVA) and ns, not significant, as compared to TNF-α treated cells. Med, Medium. Bar, 20 µm.
