# Supplementary material for: Mycobacterium bovis BCG promotes tumor cell survival from tumor necrosis factor-α-induced apoptosis
Source: Mol Cancer. 2014 Sep 11;13:210. doi: 10.1186/1476-4598-13-210 (PMC4174669; doi:10.1186/1476-4598-13-210)
Supplement: Supplementary file 5 — Additional file 5: Table S1: Primers used in the study. (DOC 44 KB) [file 12943_2014_1415_MOESM5_ESM.doc]

**Additional file 5: Table S1**

**Additional file 5: Table S1. Primers used in the study.**

| Sr. No. | Gene name | Sequence |
| --- | --- | --- |
| **Quantitative real time RT-PCR** | | |
| 1 | *GAPDH* forward | 5’-ggagcgagatccctccaaaat-3’ |
|  | *GAPDH* reverse | 5’-ggctgttgtcatacttctcatgg-3’ |
| 2 | *CDKN1A* forward | 5’-cctgtcactgtcttgtaccct-3’ |
|  | *CDKN1A* reverse | 5’-gcgtttggagtggtagaaatct-3’ |
| 3 | *NOXA* forward | 5’-gtgcccttggaaacggaaga-3’ |
|  | *NOXA* reverse | 5’-ccagccgcccagtctaatca-3’ |
| 4 | *PUMA* forward | 5’-cagactgtgaatcctgtgct-3’ |
|  | *PUMA* reverse | 5’-acagtatcttacaggctggg-3’ |
| 5 | *COP-1* forward | 5’-aacgtgctattataccaaggctg-3’ |
|  | *COP-1* reverse | 5’-tcagtggtctatactctcctgtc-3’ |
| 6 | *SHH* forward | 5’-ctcgctgctggtatgctcg-3’ |
|  | *SHH* reverse | 5’-atcgctcggagtttctggaga-3’ |
| 7 | *GLI1* forward | 5’-agcgtgagcctgaatctgtg-3’ |
|  | *GLI1* reverse | 5’-cagcatgtactgggctttgaa-3’ |
| 8 | *GLI2* forward | 5’-ctgcctccgagaagcaagaag-3’ |
|  | *GLI2* reverse | 5’-gcatggaatggtggcaagag-3’ |
| 9 | *PTCH1* forward | 5’-ccagaaagtatatgcactggca-3’ |
|  | *PTCH1* reverse | 5’-gtgctcgtacatttgcttggg-3’ |
| 10 | *SMO* forward | 5’-tcgaatcgcta`ccctgctg-3’ |
|  | *SMO* reverse | 5’-caagcctcatggtgccatct-3’ |
| **Chromatin immunoprecipitation** | | |
| 1 | 28S rRNA forward | 5’-ctgggtataggggcgaaagac-3’ |
|  | 28S rRNA reverse | 5’-ggccccaagacctctaatcat-3’ |
|  | **For GLI1 binding** | |
| 2 | *COP-1* forward | 5’-tagagacggggtttcgctatc-3’ |
|  | *COP-1* reverse | 5’-gcacaaaacaaggaaacctag-3’ |
